# SFCE-RAPIRI Phase I Study of Rapamycin Plus Irinotecan: A New Way to Target Intra-Tumor Hypoxia in Pediatric Refractory Cancers

**DOI:** 10.3390/cancers12103051

**Published:** 2020-10-20

**Authors:** Sarah Jannier, Véronique Kemmel, Consuelo Sebastia Sancho, Agathe Chammas, Amelia-Naomie Sabo, Erwan Pencreach, Françoise Farace, Marie Pierre Chenard, Benoit Lhermitte, Birgit Geoerger, Isabelle Aerts, Didier Frappaz, Pierre Leblond, Nicolas André, Stephane Ducassou, Nadège Corradini, Anne Isabelle Bertozzi, Eric Guérin, Florence Vincent, Michel Velten, Natacha Entz-Werle

**Affiliations:** 1 Pediatric Onco-Hematology Unit, University Hospital of Strasbourg, 67098 Strasbourg, France; Sarah.jannier@chru-strasbourg.fr (S.J.); Florence.vincent@chru-strasbourg.fr (F.V.); 2 Laboratory of Biochemistry, University Hospital of Strasbourg, 67098 Strasbourg, France; Veronique.kemmel@chru-strasbourg.fr (V.K.); amelia-naomi.sabo@chru-strasbourg.fr (A.-N.S.); Eric.guerin@chru-strasbourg.fr (E.G.); 3 Laboratory of Pharmacology and Toxicology in Neurocardiology-EA7296, University of Strasbourg, 67000 Strasbourg, France; 4 Radiology Department, Pediatric Unit, University Hospital of Strasbourg, 67098 Strasbourg, France; Consuelo.sebastiasancho@chru-strasbourg.fr (C.S.S.); Agathe.chammas@chru-strasbourg.fr (A.C.); 5 Oncobiology Platform, Laboratory of Biochemistry and Molecular Biology, University Hospital of Strasbourg, 67098 Strasbourg, France; erwan.pencreach@chru-strasbourg.fr; 6 «Circulating Tumor Cells» Translational Platform, Gustave Roussy, University of Paris-Saclay, 94800 Villejuif, France; francoise.farace@gustaveroussy.fr; 7 Pathology Department, University Hospital of Strasbourg, 67098 Strasbourg, France; Marie-Pierrette.CHENARD@chru-strasbourg.fr (M.P.C.); benoit.lhermitte@chru-strasbourg.fr (B.L.); 8 Centre de Ressources Biologiques, University Hospital of Strasbourg, 67098 Strasbourg, France; 9 Gustave Roussy Cancer Center, Department of Pediatric and Adolescent Oncology, Université Paris-Saclay, INSERM U1015, 94800 Villejuif, France; birgit.geoerger@gustaveroussy.fr; 10 Oncology Center SIREDO, Institut Curie, PSL Research University, 75005 Paris, France; isabelle.aerts@curie.fr; 11 Pediatric Oncology Department, Léon Berard Institute, 69373 Lyon, France; didier.frappaz@ihope.fr (D.F.); pierre.leblond@ihope.fr (P.L.); nadege.corradini@ihope.fr (N.C.); 12 Pediatric Oncology Unit, Oscar Lambret Center, 59020 Lille, France; 13 Pediatric Onco-Hematology Unit, CHU La Timone, 13005 Marseille, France; nicolas.andre@ap-hm.fr; 14 Pediatric Onco-Hematology Department, University Hospital of Bordeaux, 33000 Bordeaux, France; stephane.ducassou@chu-bordeaux.fr; 15 Pediatric Oncology Unit, University Hospital of Nantes, 44093 Nantes, France; 16 Pediatric Onco-Hematology Department, University Hospital of Toulouse, 31059 Toulouse, France; bertozzi.ai@chu-toulouse.fr; 17 Clinical Research Department, ICANS, 67200 Strasbourg, France; michel.velten@unistra.fr; 18 UMR CNRS 7021, Laboratory Bioimaging and Pathologies, Tumoral Signaling and Therapeutic Targets, Faculty of Pharmacy, 67401 Illkirch, France

**Keywords:** intra-tumor hypoxia, mTor, HIF1, pediatric refractory cancers

## Abstract

**Simple Summary:**

More and more relapsing or refractory pediatric cancers are described to present hypoxic features linked to a worse outcome. Therefore, the aim of our phase I study RAPIRI was the targeting of the central node mTor/HIF-1α with rapamycin plus irinotecan and determine the appropriated dose of this combination. As expected, the tolerance was optimal across all dose levels and no maximum tolerated dose of both drugs was reached. The pharmacokinetics (PK) helped us to refine the doses to use in the future phase II trial and the importance of PK follow-up in such combination. We also confirmed in almost half of the interpretable patients for tumor response a non-progressive disease. All those observations additionally to the ancillary’s studies provide strong evidence to propose a next trial focusing on brain tumors and sarcomas and using biweekly 125 mg/m^2^ irinotecan dose with a PK follow-up and a rapamycin dose of 1.5 mg/m^2^/day, reaching a blood concentration above 10 µg/L.

**Abstract:**

Hypoxic environment is a prognostic factor linked in pediatric cancers to a worse outcome, favoring tumor progression and resistance to treatments. The activation of mechanistic Target Of Rapamycin (mTor)/hypoxia inducible factor (HIF)-1α pathway can be targeted by rapamycin and irinotecan, respectively. Therefore, we designed a phase I trial associating both drugs in pediatric refractory/relapsing solid tumors. Patients were enrolled according to a 3 + 3 escalation design with ten levels, aiming to determine the MTD (maximum tolerated dose) of rapamycin plus irinotecan. Rapamycin was administered orally once daily in a 28-day cycle (1 to 2.5 mg/m^2^/day), associating biweekly intravenous irinotecan (125 to 240 mg/m^2^/dose). Toxicities, pharmacokinetics, efficacy analyses, and pharmacodynamics were evaluated. Forty-two patients, aged from 2 to 18 years, were included. No MTD was reached. Adverse events were mild to moderate. Only rapamycin doses of 1.5 mg/m^2^/day reached over time clinically active plasma concentrations. Tumor responses and prolonged stable disease were associated with a mean irinotecan area under the curve of more than 400 min.mg/L. Fourteen out of 31 (45.1%) patients had a non-progressive disease at 8 weeks. Most of them were sarcomas and brain tumors. For the phase II trial, we can then propose biweekly 125 mg/m^2^ irinotecan dose with a pharmacokinetic (PK) follow-up and a rapamycin dose of 1.5 mg/m^2^/day, reaching a blood concentration above 10 µg/L.

## 1. Introduction

Intra-tumor hypoxia, present in multiple solid tumors, induces usually aberrant microvasculature and tumor necrosis [1,2,3,4,5,6,7]. It is also known to increase genetic instability through apoptosis and decreased DNA repair capacities, promoting a metastatic and highly chemo- and radio-resistant tumor cell phenotype, like in pediatric brain tumors, sarcomas, or neuroblastomas. This process has been described as a marker of worst prognosis [1,2,3]. In order to preserve cell proliferation in this hypoxic microenvironment, cancer cells are promoting the upregulation of mechanistic Target Of Rapamycin (mTor) and subsequently are stabilizing hypoxia inducible factors (HIFs) to induce a cellular adaptation to the decrease of oxygen concentration [8,9,10,11]. HIF-1α accumulation is translocating into the nucleus to increase transcription of downstream signaling, leading to neoangiogenesis, metabolic switch, cell propensity to migrate, and tumor cell stemness features. Then, when inhibiting these proteins, the idea is to prevent the effects of hypoxic signals and subsequently stop cancer cell adaptation, tumor growth, and metastatic evolution.

Several studies have showed that tumors, like neuroblastoma, rhabdomyosarcoma, high-grade glioma, or osteosarcoma, are overexpressing these targets and are sensitive to mTor inhibition [4,5,12,13,14,15,16,17,18,19]. Therefore, rapamycin has been used in multiple phase I trials in these malignancies as single agent or in combination with chemotherapy [12,13,14,15,16,17,18,19,20,21]. The results of these protocols were promising, but they also showed limitations due to a positive feedback loop, reactivating constantly mTor through upstream RAS/mitogen-activated protein kinase (MAPK) pathway [22,23,24,25]. In our Lab, a previous work on colon cancers showed a high inhibition of HIF-1α with irinotecan in microenvironments varying oxygen concentrations from 5 to 1% [26,27]. This drug, classically considered to be a DNA-damaging agent, was downregulated HIF-1α target genes along with a stark reduction of HIF-1α protein in the xenografted colon cancers. In our preclinical work on pediatric cancers (e.g., high-grade glioma cell lines [25] and unpublished data on pediatric patient-derived osteosarcoma cell lines), targeting the central node mTor/HIF-1α with rapamycin and irinotecan was resulting in anti-proliferative effects, as well as a metabolic impact on tumor cells. In western blot and transcriptomic analyses, we showed that the drug combination was suppressing concomitantly HIF-1α and mTorc1 expressions. When this maximal attenuation of both proteins was present, we were also able to avoid downstream and upstream signaling pathway upregulation with a less oncogenic addiction to RAS/MAPK signal [25]. We observed in our in vitro experiments under hypoxic atmospheres a synergistic effect with this double strategy in cell lines overexpressing the phosphor-mTor and HIF-1α proteins but not in cells expressing EPAS1/HIF-2α additionally to those targets [25]. With this preclinical background, we designed in the SFCE (Société Française des Cancers de l’Enfant) committee of new drugs and pharmacology a phase I study in pediatric refractory or recurrent cancers with increasing doses of rapamycin and irinotecan to target in concomitantly mTorc1 and HIF-1α. The treatment scheme was based on unpublished in vitro and in vivo (subcutaneous xenografted mouse) work on high-grade glioma cell lines where daily repeated doses of rapamycin was inhibiting mTorc1 constantly and dose spacing of irinotecan every 2 or 5 days was able to inhibit profoundly HIF-1α protein expression. So, the subsequent patient administration schedule was translated in daily rapamycin intake and intra-venous irinotecan every 2 weeks. It resembles the treatment administration usually done in protocols, including bevacizumab and irinotecan, in adult high-grade gliomas [28]. Up to date, no other pediatric trial was using this association and schedule in advanced pediatric tumors with the idea of hypoxia targeting and based on such preclinical work.

To go further, this phase I trial, named RAPIRI (RAPamycin plus IRInotecan), was launched in 9 centers in France and comprised 10 levels of drug escalation. The primary endpoint of this trial was to determine the MTD (maximum tolerated dose) of this combination in pediatric patients. A secondary endpoint was to determine toxicities and PK (pharmacokinetic) profiles of both molecules during the first treatment cycle. We were expecting high PK variabilities between the 2 drugs, as they are both metabolized by intrahepatic cytochrome P450 (CYP) enzymes and might be demonstrated competitive inhibition. For a better understanding of potential toxicities linked specifically to irinotecan, a polymorphism analysis of *UGT1A1* (Uridine diphosphate-Glucuronosyl Transferase 1A1) was also added. In fact, SN-38, which is an active metabolite of irinotecan and measured in our trial, is excreted after conjugation by *UGT1A1* to form the inactive compound SN-38G. Variants of this *UGT1A1* gene exist and, in patients with certain polymorphisms, its glucuronidation is impaired, resulting in a risk increase of serious adverse reactions to irinotecan [29,30,31]. Wild-type *UGT1A1* is characterized by 6 TA (Thymidine Adenine) repeats in the promotor region, whereas UGT1A1*28 (rs8175347) carriers have an extra TA repeat that impairs *UGT1A1* transcription and, thereby, reduces expression by approximately 70% in homozygous genotypes. UGT1A1*6 also results in a reduction of *UGT1A1* activity in individuals carrying the heterozygous UGT1A1*6/*28 genotype. So, both homozygous (7/7 TA) and heterozygous genotypes (6/7 TA) are usually linked to a higher risk of irinotecan toxicity [29]. In addition to these PK analyses, the efficacy endpoint of the combination at 2 cycles was aimed to determine for next study the appropriate doses of rapamycin and irinotecan. The PDics (pharmacodynamics) was designed to understand unresponsiveness to this strategy and focused on hypoxia radiological parameters and on immunohistochemical and cytokine/chemokine profiling. To approach imaging hypoxic features and based on previous publications, we measured on Magnetic Resonance Imaging (MRI) diffusion acquisitions the ADC (Apparent Diffusion Coefficient) [32,33,34,35,36]. This MRI parameter was mostly studied in adult cancers and in brain tumors, where, on ADC maps, the decrease of ADC mean values was significantly linked to tumor hypoxia and HIF-1α hyperexpression at diagnosis [32,33,34,35]. In our own experience, we were able in another pediatric high-grade gliomas’ study to correlate this low ADC mean value to an hyperexpression of HIF-1α and/or HIF-2α and patient worst outcome [34]. Therefore, it was only performed in brain tumors included in RAPIRI trial, which were all followed by MRI imaging. The immunohistochemical profiling was investigating on tumor sample at diagnosis the 2 targets: activated phospho-mTor and HIF-1α, but also phospho-Akt, which is upstream to mTorc1 and stimulating its expression, and HIF-2α, which was underlined as a biomarker of treatment resistance in our in vitro preclinical work [25]. We ended by cytokine/chemokine profiling during cycle 1 to explore plasmatic pro-inflammatory cytokines and angiogenic factors and study their modulation in our treated patients [37,38,39]. In fact, many cytokines and angiogenic factors are hypoxia regulated. We hypothesized that a profile of blood-based biomarkers would correlate with tumor response and might be practical for monitoring during treatment.

## 2. Results

### 2.1. Patients’ Characteristics

**Figure 1:**
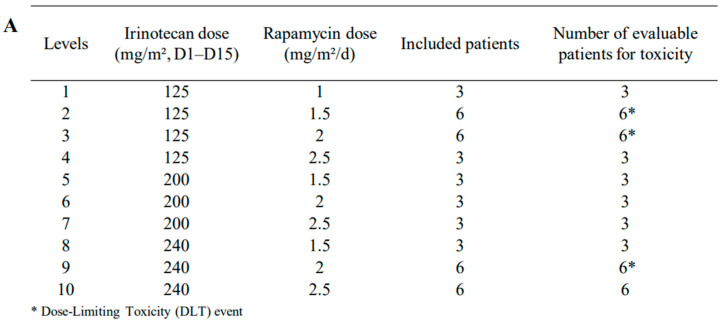

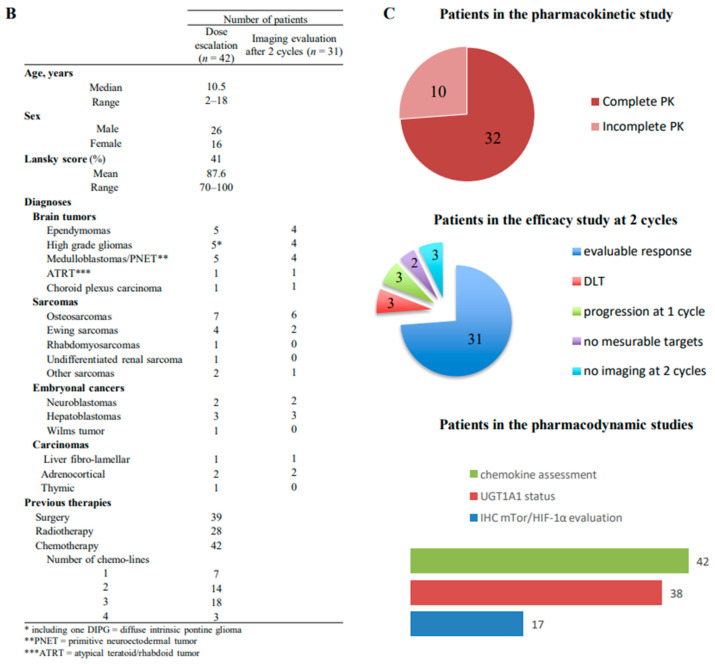
Summary of RAPIRI (RAPamycin plus IRInotecan) Phase I characteristics with levels, doses, and patients’ numbers (**A**), patients’ demographics, and clinical characteristics (**B**), and the ancillary’s studies (**C**).

Forty-two patients were enrolled from March 2011 to November 2013. Patients’ demographics and clinical characteristics are described in Figure 1A,B. All were fully eligible for toxicity evaluation. The median age at enrollment was 10.5 years with a predominance of males (sex ratio: 1.62). The most common tumor types were brain cancers (n = 17) and sarcomas (n = 15). All patients received at least one cycle and the median duration of treatment was 2 cycles. Eight patients achieved completely the six planned cycles of the study and one could extend to a total of 8 cycles. Most of the patients were heavily pretreated with 2 or more previous chemotherapeutic lines. Twenty-eight patients had a previous radiotherapy. In Figure 1C, we detailed the numbers of patients evaluable for PK (32 patients) and tumor response (31, 34 if including the three early progressions at 1 cycle). For PDics studies, all patients had cytokine/chemokine measures at D0, D15, and 30 of the first cycle. The immunohistochemical assessment was performed in 17 patients, where FFPE (formalin-fixed, paraffin-embedded) diagnostic samples were available, and 38 children were assessed for *UGT1A1* polymorphism. The ADC calculation was performed in the 17 enrolled brain cancers on their inclusion MRI imaging.

### 2.2. Dose Escalation and Toxicity

All dose levels are provided in Figure 1A. A dose-limiting toxicity (DLT) was reported for one patient in dose levels 2, 3, and 9. Nevertheless, during extension to 6 patients per level, no other patient experienced a DLT and, then, no MTD (maximum tolerated dose) was identified. Among the 3 DLTs, one patient with undifferentiated renal sarcoma included in dose level 2 had grade 3 diarrhea and grade 4 infection (septic shock). The patient in dose level 3 experienced a grade 3 anorexia and grade 3 oral mucositis. The patient at dose level 9 had grade 3 diarrhea. All those toxicities resolved without sequela. These three patients discontinued their treatment definitely.

**Figure 2:**
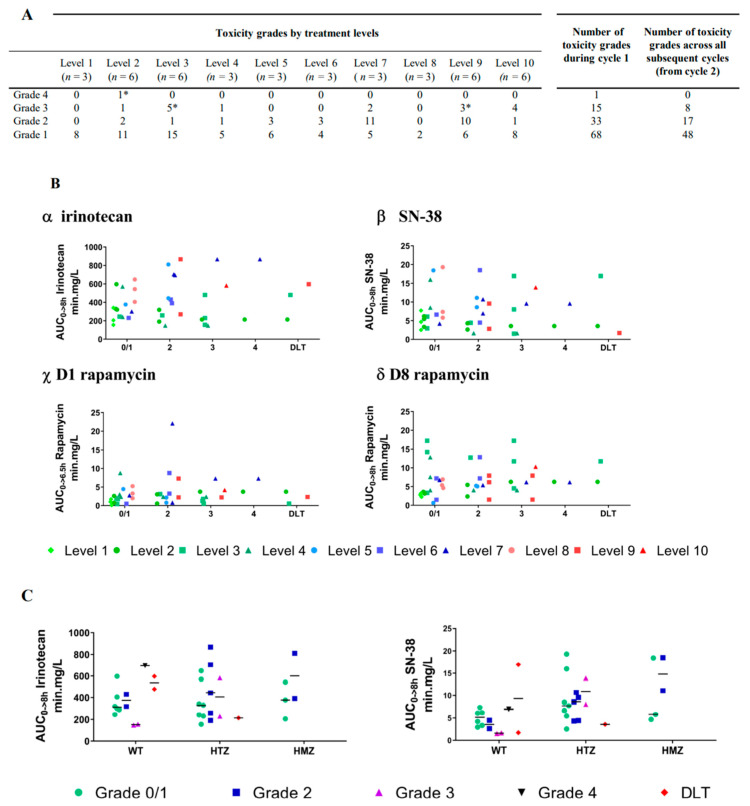
Summary of the toxicity evaluation. (**A**) Reported toxicity grades according to dose escalation during cycle 1 and across all subsequent cycles. (* is underlining each dose-limiting toxicity (DLT) event reported at levels 2, 3, and 9). (**B**) Mean AUCs (area under the curves) for irinotecan (α), SN-38 (β), rapamycin day 1 (D1) (χ) and day 8 (D8) (δ) during cycle 1 according to toxicity grades and DLT. Thirty-nine dots are observed. Most of the 32 patients with pharmacokinetic (PK) evaluation had 2 or more adverse events (AEs) of the same grade and are presented as a single dot. Seven additional dots are illustrating AEs of different grades for a same patient. (**C**) Mean AUCs for irinotecan (α) and SN-38 (β) in each toxicity grades according to Uridine diphosphate-Glucuronosyl Transferase 1A1(*UGT1A1*) polymorphism.

During cycle 1, 35 among the 42 treated children (83.3%) experienced reversible AE (adverse events) related to study treatments. Twenty-five patients had at least 2 concomitant AEs during this first cycle. One hundred and seventeen AEs were described during cycle 1 and are listed in Figure 2A and Figure S1. There were predominantly grade 1 (68/117) and 2 (33/117) events. The most common drug-related AEs were vomiting, diarrhea, nausea, oral mucositis, and abdominal pain. Only 6 patients during cycle 1 presented grade 3 hematological toxicities that resolved before starting cycle 2 (Figure S1). Three other patients were diagnosed for a grade 3 hypophosphoremia, a grade 3 hypokaliemia, or a grade 3 ALP (alkaline phosphatase) increase (Figure S1). None of these grade 3 toxicities required dose reduction in the subsequent cycles. No significant associations were established between dose levels, number of cycles, and grades of AEs or between PK, AE grades, and dose levels (Figure 2B). An increase of mean AUC (area under the curve) of irinotecan, SN-38, or D1 rapamycin PK was not linked to higher grades of AE, except for the patients with high concentrations of rapamycin at D8. Finally, we were only able to correlate an increase of toxicity grades to a lower BMI (Body Mass Index) (*p* = 0.0701) (Figure S2A) and to a Lansky or Karnofsky score decrease during first cycle (*p* = 0.0079) (Figure S2B).

Regarding irinotecan toxicity and AUC’s variabilities, we tried to correlate *UGT1A1* polymorphism to PK assessment and AE grades. Among the 38 patients where we were able to determine *UGT1A1* polymorphism, 5 patients were homozygous (13.2%), 18 patients were heterozygous (47.3%), and 15 patients were wild-type (39.5%). No significant correlation was determined between irinotecan and/or SN-38 AUC increase and *UGT1A1* subgroups (Figure 2C). Surprisingly, 2 of the 3 DLTs were observed in the wild-type *UGT1A1* subgroup and one in a heterozygous patient. Significant metabolic differences were observed among *UGT1A1* subgroups (Figure S3). In fact, the accumulation of SN-38, illustrated in Figure S3 by the decrease of the ratio irinotecan/SN-38 AUC, is significantly correlated with a lower activity of *UGT1A1* in heterozygous and homozygous subgroups (*p* = 0.0166 and *p* = 0.0011, respectively). In fact, the wild type group showed a ratio for irinotecan/SN-38 AUCs of 82.2, and homozygous and heterozygous subgroups had a ratio of 49.2 and 50.8, respectively. This is consistent with a lower metabolism of SN-38 and a normal carboxyesterase activity [29]. Nevertheless, SN-38 accumulation was not sufficient to explain its significant role in patient toxicities, as illustrated in Figure 2C.

### 2.3. Pharmacokinetics (PK)

**Figure 3:**
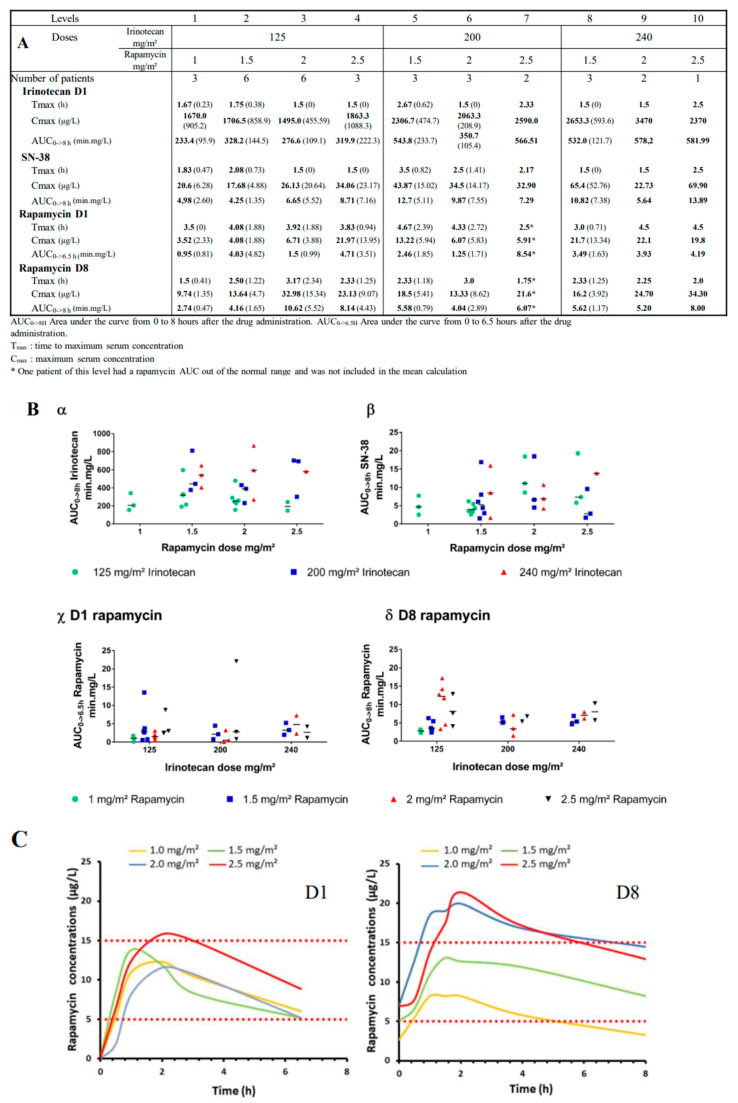
Pharmacokinetics of irinotecan, its metabolite SN-38, and rapamycin during cycle 1. (**A**) Global pharmacokinetic parameters (means and standard deviations) for each parameter. (**B**) AUCs (area under the curves) of irinotecan (α) and SN-38(β) linked to rapamycin escalation doses and AUCs of rapamycin at day 1 (D1) (χ) and day 8 (D8) (δ) linked to irinotecan escalation doses. (**C**) Rapamycin concentration-time profiles in the different dose levels of rapamycin at D1 (left side) and at D8 (right side).

The complete PK measures, done in 32 patients during cycle 1 of treatment, are summarized in Figure 3A. C_max_ (maximum concentration) and AUC_0-8H_ of irinotecan tend to increase slightly with irinotecan dose escalation from 125 to 240 mg/m^2^, but without any statistical correlation (R^2^ = 0.39) (Figure 3A). As expected, and globally, irinotecan AUCs varied from 147.2 to 867.2 min.mg/L, but mean AUCs of irinotecan per irinotecan dose level appeared stable independently from rapamycin doses (Figure 3(Bα)). For SN-38 AUCs, no correlation was identified between SN-38 AUCs and irinotecan doses (R^2^ = 0.004), nor with irinotecan AUCs (R^2^= 0.003) or rapamycin doses (R^2^ = 0.07) (Figure 3B). Despite a global high variability of rapamycin AUCs (Figure 3(A,Bγ,Bδ)), the mean AUCs of rapamycin at D1 and D8 remained stable independently from irinotecan doses, but increased slightly with rapamycin dose escalation from 1 to 2.5 mg/m^2^. Surprisingly, three patients had three to tenfold higher rapamycin AUCs. The only associated treatment given to those 3 patients was cotrimoxazole, a light enzymatic inhibitor (CYP2C8 and CYP2C9). Nevertheless, this impact was not detected in the 16 patients also treated with cotrimoxazole. Globally, the D8 rapamycin concentrations were not significantly different for patients treated with or without cotrimoxazole (6.75 µg/L vs. 5.3 µg/L, respectively, *p* = 0.22). Additionally, nine patients remained with very low T_0_ rapamycin concentrations at D8, between 0.66 and 3.0 µg/L. Three of them were treated with 1 mg/m^2^/day, two with 1.5 mg/m^2^/day, three with 2 mg/m^2^/day and one with 2.5 mg/m^2^/day. Only 2 out of 9 had concomitant oxcarbazepine treatment, which is a CYP3A4 inductor and a CYP2C19 inhibitor. In the seven remaining patients no explanation was explaining the low rapamycin values. In this subgroup of low rapamycin concentrations, 4 patients had also lower irinotecan and SN-38 AUCs.

Despite this high range of variability, when looking closely to rapamycin concentrations over time in Figure 3C, the dose of 1.5 mg/m^2^/day on D1 and D8 was the only one reaching normal concentration ranges considered as clinically active (e.g., 5–15 µg/L) along administration time. Therefore, to obtain a balance between efficacy and a safe ratio risk/benefit for the patients, it might be the dose to use in combination with irinotecan. For irinotecan doses, looking back to the mean AUCs per toxicity grades or DLT in Figure 2A, we had grade 3 and 4 toxicities until the level 2 at 125 mg/m2/dose and with variable AUCs of irinotecan and SN-38. At least, levels 1 and 8 showed only grade 1 events, but a variable AUC range for irinotecan or SN-38. Nevertheless, the average of irinotecan AUCs was between 300 and 600 min.mg/L in a majority of levels and, especially, when rapamycin was used at 1.5 mg/m^2^/day.

### 2.4. Anti-Tumor Effect, Links with PK, and Groups of Tumors

**Figure 4:**
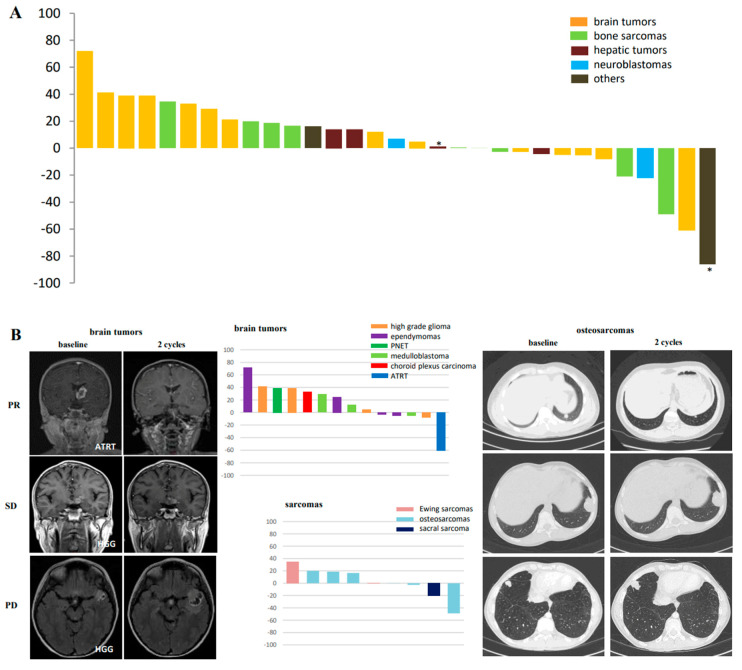

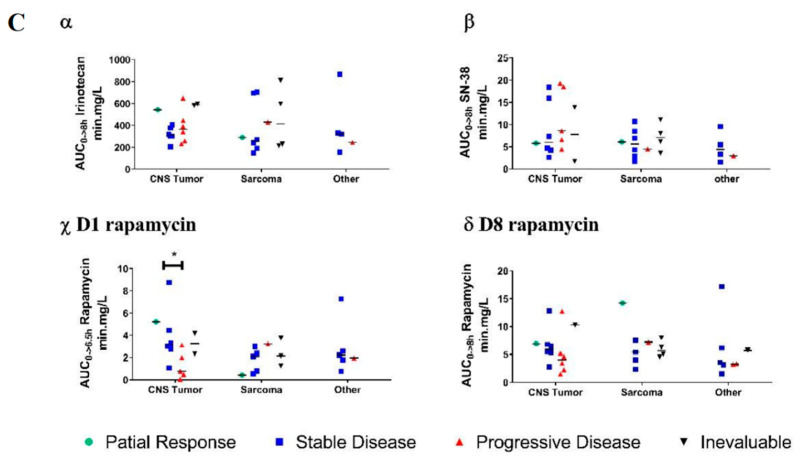
Efficacy assessment at 2 cycles of treatment. (**A**) Tumor response after 2 cycles based on Response Assessment in Neuro-Oncology (RANO) or Response Evaluation Criteria In Solid Tumors (RECIST) 1.1 criteria in 31 evaluable patients. (* patients with largest target response but new pulmonary lesions). (**B**) Examples of brain tumors and bone sarcomas’ responses (partial response (PR), stable disease, and progression disease (PD). (**C**) Mean AUCs for irinotecan (α), SN-38 (β), rapamycin day 1 (D1) (χ) (* *p* < 0.05) and day 8 (D8) (δ) according to tumor subgroups.

Thirty-one out of 42 enrolled children were eligible for tumor response evaluation at 2 cycles (Figure 1C and Figure 4A) on target lesions comparatively to baseline. Three patients had an early progressive disease (PD) at 1 cycle with radiological assessment and were integrated in the PD subgroup. They are not presented in Figure 4A.

Two patients had a confirmed PR (partial response) persisting over 4 cycles of treatment: one with an ATRT (Atypical Teratoid Rhabdoid Tumor) and one with a pulmonary metastatic osteosarcoma. They were treated at dose level 3 and 8 (Figure 4A,B) and received 6 and 5 cycles, respectively. An SD (Stable disease) was observed in 19 patients: 7 brain tumors, 6 sarcomas, 2 hepatoblastomas, 2 neuroblastomas, 1 fibro-lamellar hepatocarcinoma, and 1 adrenocortical carcinoma. Nine of them had a persistent SD at 4 cycles, and 6 out of the nine children reached the 6th cycle. One additional hepatoblastoma and one adrenocortical carcinoma had a very good response on the tumor target (they are marked by * on Figure 4A), but new millimetric lesions in the lung appeared after 2 cycles. Including those two and the 3 early PD at 1 cycle, we finally had a total of 13 PDs. Nevertheless, 14 out of 31 (45.1%) patients had a non-progressive disease at 8 weeks of treatment, and 11 out of 31 (35.5%) at 16 weeks.

To go further and see if PK results might be a key in tumor responses, we investigated correlation between tumor responses at 2 cycles and dose levels and determined the AUCs per response subgroups. No correlation between tumor responses and dose escalation was observed (e.g., Figure S4) (*p* = 0.65). For the AUCs, the two PRs, who were treated in level 3 and 8, had a mean AUC of irinotecan of 412 min.mg/L (290–594) and a mean AUC of D8 rapamycin of 10.55 min.mg/L (6.9–14.6), which is consistent with a range of concentration known to be clinically active as already described above in Figure 3C. The mean irinotecan AUCs in patients with SD was 358.2 min.mg/L (147–709) with a mean AUC of D8 rapamycin of 9.2 min.mg/L (2.1–73). In the PD subgroup, the mean AUC of irinotecan was 321 min.mg/L and of D8 rapamycin was 6.5 min.mg/L. Ten out of the 19 stable tumors were treated in the four first levels, where irinotecan 125 mg/m^2^/dose was administered. When looking to the patients with target lesion shrinkage from 0 to −86%, the irinotecan mean AUC was 404.2 mg.min/L and, when we were considering disease control at 8 weeks, it was 396.16 mg/min/L and, for responders or stabilized patients at 16 weeks, we had 405.1 mg.min/L. However, no statistically significance was evidenced.

Finally, we studied the RAPIRI treatment efficacy depending on tumor types. In patients with brain tumors, SD at cycle 2 was observed in 3 ependymomas, 2 high grade gliomas, and 2 medulloblastomas and we obtained a confirmed PR in an ATRT (Figure 4B). All had a mean number of cycles above 5 (e.g., 5.7) and had a prolonged disease control even when they were heavily pretreated. The mean AUCs of each compound might be higher in responding tumors (Figure 4C) and clearly significant for the mean AUC of D1 rapamycin (*p* = 0.03). For irinotecan AUC, it was only a tendency (*p* = 0.08). For patients with sarcomas, 4 out of 6 osteosarcomas had an SD and one a PR (Figure 4B). Only one Ewing sarcoma had an SD. We were not able to have a significant correlation with mean AUC of each drug (*p* = 0.36 and *p* = 0.82).

### 2.5. PDics Studies Were Able to Underline Biomarkers of Tumor Resistance to Hypoxia Targeting

**Figure 5:**
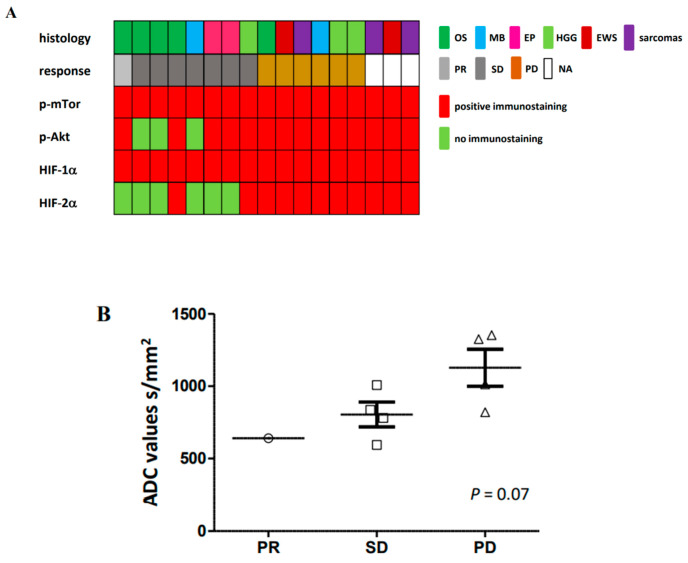
Pharmacodynamic evaluations considering protein expression profiling by immunohistochemistry. (**A**) Results of phospho-mechanistic Target Of Rapamycin (p-mTor), phospho-Akt (p-Akt), hypoxia inducible factor (HIF)-1α, and HIF-2α in 17 tumors where 1 partial response (PR), 7 stable disease (SD), 6 progressive disease (PD), and 3 unevaluable (Not available = NA) patients for response are described) and imaging assessment with ADC (Apparent Diffusion Coefficient) evaluations in brain tumor population on DWI (diffusion weighted imaging) baseline sequences. (**B**) The calculated mean ADC per group of tumor responses was slightly linked to a probable better response to RAPIRI trial in case of lower ADC values (*p* = 0.07).

To understand responsiveness and resistance to this combination, as well as to warrant some hypoxic biomarkers involved in the tumors, we, first, studied protein expression profiles in tumors where diagnostic FFPE samples were available. The choice of biomarkers was guided by the targeted therapies we used on mTorc1 and HIF-1α and the upstream and parallel signaling represented by p-Akt (phospho-Akt) and HIF-2α. We were able in 17 patients to study their diagnostic samples and, among all biomarkers, correlated significantly the HIF-2α hyperexpression by immunohistochemistry with PD subgroup (Figure 5A) (*p* < 0.005). Examples of these stainings are presented in Figure S5 for each response group comparatively to a positive control. All tumors were expressing both targeted proteins (e.g., activated phospho-mTor and HIF-1α) independently from response to study treatment. Except 3 tumors (two osteosarcomas and one medulloblastoma), all diagnostic specimens were also presented an activation of upstream signaling with p-Akt protein hyperexpression. So, a majority of the assessed patients had a probable hypoxic-driven protein profile and were able to attenuate it through the RAPIRI trial. Nevertheless, no biopsies were required at the end of treatment, and we did not have the opportunity to have patients with complementary surgical approaches to this protocol treatment.

To go further in the assessment of hypoxic tumor microenvironment, we performed in brain tumors mean ADC calculation on ADC maps established on DWI (diffusion weighted imaging) of baseline MRIs. Figure 5B shows a statistical trend between a lower ADC value and PR or SD patients (*p* = 0.07). All PD patients were presenting higher ADC values (>1000 s/mm^2^). We already know that a lower ADC value was mostly able to predict a higher hypoxic environment in the tumor, but, with the small number of patients, where we had availability of diagnostic samples, we were not able to do significant correlations with immunohistochemical analyses and ADC values [32,33,34,35,36].

**Figure 6:**
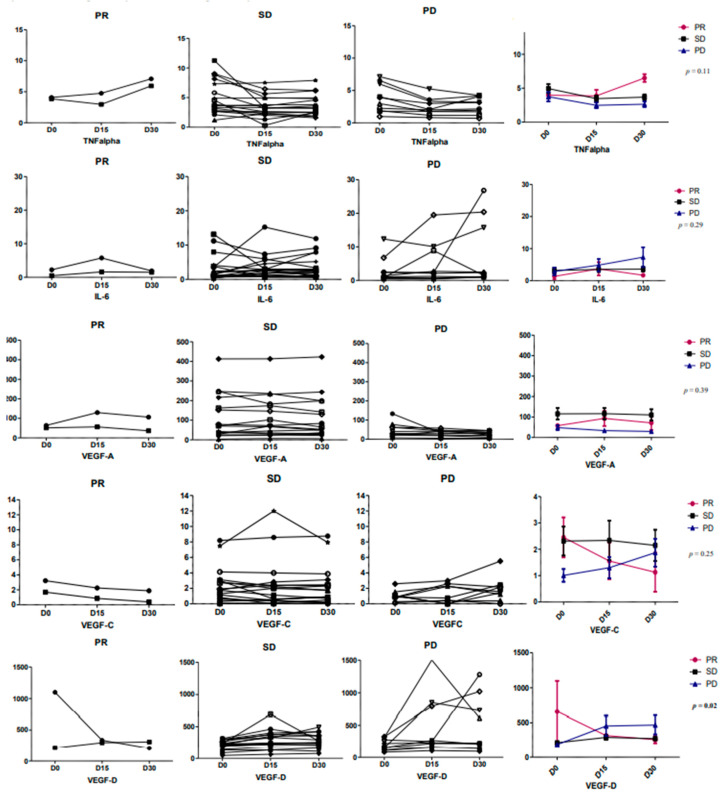
Chemokine and cytokine profiles based on tumor responses at D0, D15, and D30 of cycle 1. The Vascular Endothelial Growth factor (VEGF)-D secretion was mostly decreasing during first cycle in the partial responders and increasing in the progressive disease with a statistical significance (*p* = 0.02). The other cytokines/chemokines, like tumor necrosis factor-α (TNF)alpha, interleukine (IL)-6, and VEGF-C seems to have opposite behaviors in the partial responders and progressive disease but without any statistical significance. In all stable disease, the cytokines are globally stable during first cycle of treatment.

Finally, the cytokine/chemokine profiling to explore plasmatic pro-inflammatory markers (interleukine (IL)-1β, IL-6, IL-8, and TNFα (tumor necrosis factor-α)) and angiogenic factors (Vascular Endothelial Growth factors: VEGF-A, VEGF-C and VEGF-D, Tie-2, sFLT-1 (VEGF receptor 1), PIGF (Placental Growth Factor), and bFGF (Fibroblast Growth Factor)) was able to evidence only a preliminary statistical link between VEGF-D and tumor response groups (*p* = 0.02, Figure 6). We monitored these plasmatic markers at baseline D0 (day 0), at D15 (day 15), and D30 (day 30) during first cycle of treatment. At D15, there is a too high variability of the measures in all groups, which is pointed out the importance of the right time to perform these measures. The results comparing D0 (baseline), D15, and D30 for each cytokine/chemokine are detailed on Figure 6 and Figure S6. When comparing tumor response groups, we find out that patients with PRs had a VEGF-D decrease and a significant increase in PD group. This chemokine is usually interacting with VEGFR-2 and VEGFR-3, receptors activated and/or induced by HIF-1α hyperexpression [10,12,25,26]. The SD group had globally and as expected stable measures of each chemokine or cytokine. No specific prognostic profile was underlined in this small cohort of patients. At least, TNFalpha, IL-6, VEGF-A, -C and -D were the markers, where slight variations were observed between each response group and the cytokine/chemokine modulation (Figure 6).

## 3. Discussion

RAPIRI phase I is a pediatric study aiming to target the hypoxic biomarkers mTorc1 and HIF-1α with rapamycin and irinotecan, respectively. Our results showed that this combination was well tolerated without severe toxicities, despite a heavily pretreated population. All toxicities were manageable and were already described when using each compound alone [14,15,16,17,18,19,20,21,40,41,42,43]. As in multiple phase I exploring targeted therapies, the MTD was not determined in absence of significant DLT events up to the tenth level (rapamycin at 2.5 mg/m^2^/day and D1/D15 irinotecan at 240 mg/m^2^).

As expected, we had a high variability of PK for each compound, but not linked to dose escalation, nor to *UGT1A1* polymorphism [44,45]. In fact, we were able to show that both drugs had a competitive inhibition on their cytochrome detoxification and even increasing their doses, AUCs were still in the same ranges. This observation was already described in other trials when both drugs were metabolized by intrahepatic cytochrome [30,31,46].

For rapamycin, from 1.5 mg/m^2^/day levels, we warranted a clinically active range between 10 and 15 µg/L. Therefore, the starting RP2D (recommended phase 2 study dose) might be 1.5 mg/m^2^/day and could be modified to obtain a blood rapamycin range between 10 to 15 µg/L during all treatment. Regular controls of rapamycin residual concentration will help us to refine rapamycin dose. For irinotecan, PK analyses were suggesting that drug monitoring might be also a solution to limit the high variability of AUCs. We could propose as a RP2D starting dose of irinotecan 125 mg/m^2^ during first cycle and perform PK follow-up. The ideal targeted irinotecan AUC, like in our PR and SD subgroups, would be above 400 mg.min/L. The SN-38 AUCs did not help us to refine accurately the targeted dose and to explain irinotecan variability. They might be not used as an assessment in the phase II trial. Based on these PK analyses performed on D1 of first cycle, we could adapt irinotecan dose in the subsequent courses. The only limitation would be not to overcome 240 mg/m^2^/dose of irinotecan as already proposed in another trials [28,29,30,31,46]. This PK follow-up might also overcome the impact of concomitant therapies, like cotrimoxazole and/or antiepileptic drugs, as previously described in trials with those compounds [47,48].

Most of the patients, where we demonstrated early evidence of activity with this hypoxia-targeting strategy, were having brain tumors (ATRT, ependymomas, medulloblastomas, and high-grade gliomas), as well as osteosarcomas. So, a proposal of a phase II trial in those tumor types might be an innovative approach at relapse time. Our exploratory PD analyses support the fact that intra-tumor hypoxic microenvironment is the key in follow-up. They are useful tools to determine the appropriate patients to include in such therapeutic strategy. In fact, the presence of a low ADC mean value less than 1000 s^2^/m and the presence of p-mTor and HIF-1α hyperexpressions are warranted a tumor response or at least a stabilized disease. Nevertheless, recent publication might also provide new insight on how the irinotecan combined to mTor inhibitors would act independently from HIF-1α [49]. So, this patient profiling will be even more accurate in phase II trial to determine precisely the targets of this combination and understand their role in response. Adding the absence of HIF-2α hyperexpression in the inclusion criteria might refine the population for a more adequate response to this combination and avoid a subgroup of patients with a high risk of resistance. This last immunohistochemical biomarker is in accordance with our previous work on pediatric cell lines, where HIF-2α was only overexpressed in resistant lines to mTor/HIF-1α inhibition [25,34] and with our immunohistochemical results in RAPIRI phase I trial. The cytokine measures might be a blood measure easily done and to follow during the treatment administration, but it has to be confirmed as a tool to predict long-term tumor responses.

## 4. Materials and Methods

### 4.1. Patient Eligibility

The RAPIRI phase I trial (PHRC-national Cancerology 2010, HUS n°4791/N° Eudract: 2010-022329-13, NCT01282697) was launched in 9 centers in France. All patients, parents, and/or legal guardians gave informed consent for treatment and ancillary studies before inclusion according to national ethical committee and regulatory requirements. Patients from 1 to 21 years old with refractory or relapsed solid tumor after conventional and recommended therapies for relapses were eligible for RAPIRI trial. The tumors had to be previously histologically proven. Other eligibility criteria were Lansky or Karnofsky performance status ≥ 70%; life expectancy of more than 8 weeks; no radiotherapy and/or chemotherapy 4 weeks before starting protocol and no surgery 15 days prior starting treatment; adequate baseline hematologic, hepatic, or renal functions; no previous organ toxicity upper grade 2 according to NCI-CTCAE (National Cancer Institute’s Common Terminology Criteria for Adverse Events) (version 3.0). Main exclusion criteria were patients with constitutional coagulopathy associated to a high risk of bleeding or intra-tumor hemorrhage; other simultaneous malignancy; simultaneous severe disease; pregnancy; pre-treatment with a mTor inhibitor. For patients needing anticonvulsants, the dose regimen had to be stable within one week prior the start of first cycle.

### 4.2. Protocol Design and Drug Administration

This phase I trial followed a 3 + 3 design with 10 dose levels (Figure 1A). Doses were based on our in vitro [26] and in vivo (data not published) preclinical work. Rapamycin was administered orally once daily and irinotecan was given as a 90-min intra-venous infusion at Day 1 (D1) and Day 15 (D15) of each 28-day course. Rapamycin intake was at the end of irinotecan infusion and maintained every day at the same scheduled time. Cefpodoxime (8 mg/kg/day BID orally) was administered transiently starting 2 days prior to the intra-venous irinotecan administration and stopped after 5 days [49]. All patients were expected to receive at least one 28-day cycle to be evaluable for the primary endpoint, which was the determination of the MTD. Escalation to the next dose level was allowed if no DLT was observed during cycle 1 in all 3 patients of the same dose level. After cycle 1, the treatment could be continued for a total of 6 courses or until patients undergo disease progression or unacceptable toxicity or withdraw consent. Responding or stabilized patients might be treated longer until 12th course.

### 4.3. Toxicity Assessment

Patients underwent physical examinations and biological assessments at baseline and every week during the two first courses and every 2 weeks from the third course to the end of the trial. Adverse events were graded according to the NCI-CTCAE (version 3.0). The hematological DLT was determined during first cycle when appeared: a grade 4 neutropenia or a grade 4 thrombocytopenia more than 7 days, a grade 3 thrombocytopenia requiring platelet infusions or grade 2 or 3 neutropenia inducing a 14-days delay to start next course. Non-hematological DLT were any treatment-related grade 4 or 3 adverse event, or any event requiring treatment interruption less than 7 days. Exceptions were defined as the following grade 3 adverse events: nausea or vomiting sensitive to anti-emetics, ALT/AST (transaminases) elevation returning to baseline within 7 days, infection/fever lasting less than 5 days, hypophosphoremia corrected with oral supplementation. 

### 4.4. Pharmacokinetics (PK)

PK of irinotecan and SN-38, its active metabolite, were performed at D1 of cycle 1. Blood samples were collected prior to irinotecan infusion and 1.5, 2, 2.5, 3.5, 4.5, and 8 h after the end of infusion. Rapamycin was administered at the end of irinotecan infusion. The oral intake was the start of the PK study of rapamycin at D1, which followed the irinotecan PK schedule. At D8, rapamycin PK sampling was performed prior to rapamycin administration and after 0.5, 1, 1.5, 2, 4, and 8 h. Plasmatic irinotecan, SN-38, and rapamycin concentrations were measured using liquid chromatography/mass spectrometer methods [30,31,50]. Limits of detection were 0.1, 0.7, and 0.5 µg/L for rapamycin, irinotecan, and SN-38, respectively. Concentration-time curve and PK parameters were analyzed for each compound. Maximal concentrations (C_max_) and the time to reach the C_max_ (T_max_) were graphically identified and the AUC was calculated with a non-compartmental method.

### 4.5. Tumor Response

Disease evaluations were radiologically performed at baseline, at the end of the second cycle and every 2 cycles during treatment phase. At the end of treatment during follow-up, radiological assessment should be planned every 3 months. Patients with central nervous system (CNS) tumors were evaluated by MRI (magnetic resonance imaging) with gadolinium injected sequences (T1 and T2/FLAIR), DWI (diffusion-weighted imaging), perfusion sequences, and MR spectroscopy. Tumor response for those CNS tumors was based on Response Assessment in Neuro-Oncology (RANO) criteria [51,52]. The ADC (adherent diffusion coefficient) maps were retrospectively performed by two independent and experienced neuroradiologists. The ADC maps were calculated from DWI on solid tumor portion using a commercially available software. An ADC mean was obtained for each radiologist, and an average of both ADC values were calculated. ADC was calculated at baseline and at 2-cycle assessment [32,33,34,52]. Patients with other solid tumors had either a CT scan or MRI and their tumor responses were based on the Response Evaluation Criteria In Solid Tumors (RECIST 1.1). All imaging studies were reviewed by two independent radiologists.

### 4.6. Pharmacodynamics (PDics)

#### 4.6.1. UGT1A1 Genotyping

*UGT1A1* promoter polymorphism was identified using fragment size analysis. After genomic DNA extraction from peripheral blood [53], genotypes were assigned based on the number of TA repeats in each allele corresponding to a PCR product size: TA6/6 (76 bp PCR product), TA6/7 (76 and 78 bp PCR products), or TA7/7 (78 bp PCR product) were, respectively, considered as patient presenting a wild-type *UGT1A1* promoter, a heterozygous or a homozygous polymorphism form [29].

#### 4.6.2. Immunohistochemistry (IHC) on FFPE Diagnostic Tumor Samples

Antibodies were used against phospho-AKT, phospho-mTor (Cell Signaling Technology, Ozyme, St-Quentin-en-Yvelines, France), HIF-1α, and HIF-2α (Abcam, Paris, France). IHC was performed using an automated tissue staining system (Ventana Medical Systems, Inc., Tucson, AZ, USA). Two pathologists analyzed protein expression independently. The scoring combined the degree of positivity and the proportion of positive cells. More than 20% positive tumor cells and a moderate to strong intensity were defining a hyperexpressed protein in samples. Negative and positive immunostainings were assessed comparatively to controls obtained from previous study on pediatric high-grade gliomas published in 2017 [25].

#### 4.6.3. Plasmatic Cytokine Measures

Pro-inflammatory cytokines (interleukines: IL-1β, IL-6, IL-8 and tumor necrosis factor-α (TNFα)) and angiogenic factors (Vascular Endothelial Growth factors: VEGF-A, VEGF-C and VEGF-D, Tie-2, sFLT-1 (VEGF receptor 1), PIGF (Placental Growth Factor), and bFGF (Fibroblast Growth Factor)) were monitored at baseline, D15, and D29 of the first course. Serum measurements were determined using multiplex assays as follows: Human ProInflammatory II 4-Plex Ultra-Sensitive kit and Angiogenesis Panel 1 (human) kit (Meso Scale Discovery, Rockville, Maryland, USA). The measures were performed on a Sector S600 de Meso Scale Discovery, and the results were given in pg/mL. We used in each assessment standard samples provided by the manufacturer to establish measure sensitivity and be able to consider significant optical density values when they are at least twice as high as the background noise. All experiments were performed in triplicate.

### 4.7. Statistical Analyses

For all analyses and generated graphs, Graphpad Prism software (GraphPad Prism V6, San Diego, CA, USA) was used. Associations between dose, PK variables, *UGT1A1* status, tumor response, ADC values, and toxicity were established using two-tailed ANOVA test. If *p* < 0.05, a multiple t-test corrected by Hom Sidak method (α = 0.05) was applied.

## 5. Conclusions

The recent discoveries on intra-tumor hypoxia opened a large scope in pediatric cancers and demonstrated that this mechanism is probably playing a role and might be targeted in those cancers. Our preclinical work conducts us reasonably to design a 3 + 3 escalation dose phase I trial, where rapamycin and irinotecan could target mTorc1 and HIF-1α, respectively. Forty-two patients were enrolled in 3 years. No MTD was determined as this drugs’ association was safe and well tolerated with mild toxicities. With this therapeutic strategy 14 out of 31 evaluable patients were on disease control at 8 weeks of treatment. These promising efficacy and the combined PK/PD results allow us to think about building a pediatric phase II trial focusing on brain tumors and osteosarcomas. Our RP2D proposal will be to start with rapamycin at 1.5 mg/m^2^/day and biweekly irinotecan at 125 mg/m^2^/dose and monitored at first cycle administrations to be secondary adapted to reach a mean irinotecan AUC above 400 min.mg/L and a residual concentration measure of rapamycin at D8 in the range of 10 to 15 µg/L.

## Supplementary Materials

The following are available online at https://www.mdpi.com/2072-6694/12/10/3051/s1. Figure S1: Listing of the different toxicities reported during first course and subsequent cycles, Figure S2: Complementary data on patient toxicity profile with A) Toxicity grades according to the body mass index (BMI) and B) Toxicity grades according to the Lansky or Karnofsky score, Figure S3: *UGT1A1* variability correlations showing significant correlation between the *UGT1A1* status and the ratio of irinotecan/SN-38 AUCs, Figure S4: Tumor responses per dose level in RAPIRI trial: 31 evaluable patients. No correlations were observed between tumor responses and dose levels (*p* = 0.65), Figure S5: Immunohistochemical profiling analyses according to tumor responses. A positive control (a pediatric high-grade glioma) for all immunohistochemical staining is showed in first column of the figure. In other columns, an example of each response group is presented (scalebar = 20 µm), Figure S6: Cytokine evaluation according to response groups: partial response (PR), stable disease (SD) and progressive disease (PD). No statistical significance was observed for IL-1beta, IL-8, Tie-2, sFLT-1, PIGF and bFGF, where almost all response groups had the same plasmatic secretions during first cycle of treatment.
