# Supplementary material for: SFCE-RAPIRI Phase I Study of Rapamycin Plus Irinotecan: A New Way to Target Intra-Tumor Hypoxia in Pediatric Refractory Cancers
Source: Cancers (Basel). 2020 Oct 20;12(10):3051. doi: 10.3390/cancers12103051 (PMC7656302; doi:10.3390/cancers12103051)
Supplement: Supplementary file 1 [file cancers-12-03051-s001.pdf]

Supplementary Materials

	Toxicity related to irinotecan and rapamycin							
	Number of patients							
	adverse events observed during Cycle 1 by patient				adverse events observed during other cycles by patient			
Toxicity grades	Grade 1	Grade 2	Grade 3	Grade 4	Grade 1	Grade 2	Grade 3	Grade 4
**General toxicities**								
Anorexia	5	2	1 (DLT**)				1	
Weight loss	2						1	
Denutrition		1						
Fatigue	5	2			2	2	1	
Headache		1			1			
Pain		1						
Alopecia	1				2	1		
Skin rash		1			1	1		
Skin dryness	6				1			
Heat sensation	1				2			
**GI/metabolic toxicities**								
Diarrhea	7	5	2 (DLT*§)		4	3		
Nausea	5	3			7	1		
Vomiting	10	4	3		8	2		
Oral mucositis	5	3	1(DLT**)		7	3		
Abdominal pain	5	4			3	1		
Colitis			1					
Pyrosis	1				1			
Dehydration		1						
Renal failure					1			
Hypokaliemia	1		1					
Hyphosphoremia	1		1					
Hypertriglyceridemia	1				1			
Hypoalbuminemia		1				1		
ALP increase	1		1		1	1		
**Hematological toxicities**								
Thrombocytopenia	3		1		2			
Neutropenia	2	1	3				2	
Lymphopenia	1		2		1			
Anemia	1	1				1	1	
Febrile neutropenia								
**Infections**								
Septic shock				1 (DLT*)				
**Others**								
Hyperthermia		1			1			
Dyspnea	1							
Hyperpigmentation	1				1			
Conjonctivitis	1							
Hiccough		1						
Peripheral neuropathy	1				2			
Restless leg syndrome					1			
Total	68	33	15	1	48	17	8	0

*one patient having both grade 3 and grade 4 events
** one patient having 2 grade 3 events
§ one patient having grade 3 event

Figure S1. Listing of the different toxicities reported during first course and subsequent cycles.

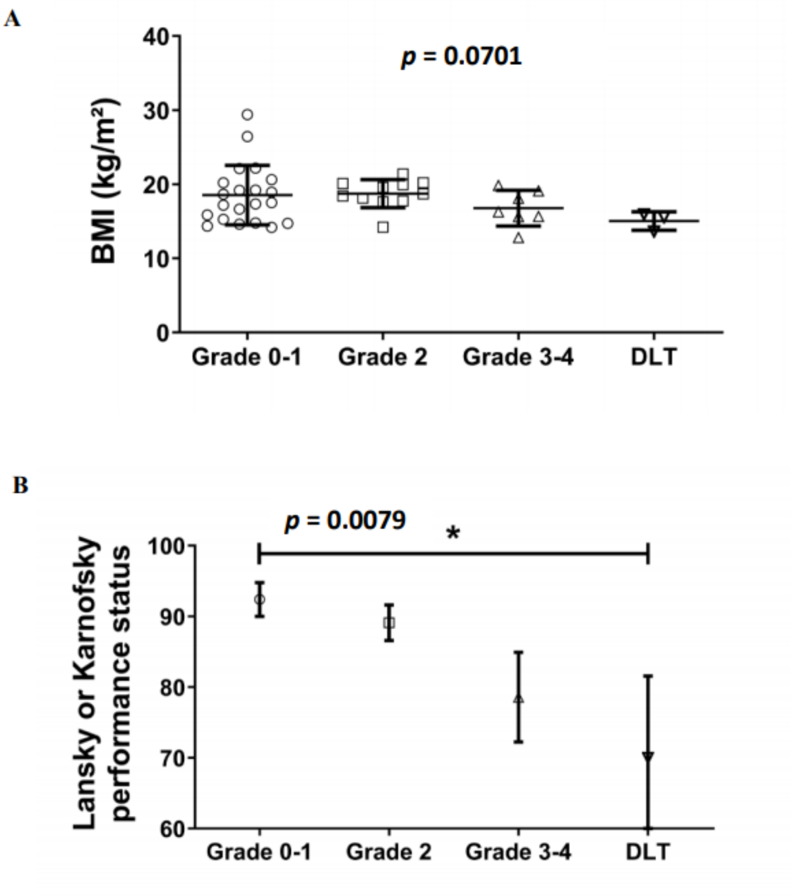

Figure S2. Complementary data on patient toxicity profile with A) Toxicity grades according to the body mass index (BMI) and B) Toxicity grades according to the Lansky or Karnofsky score.

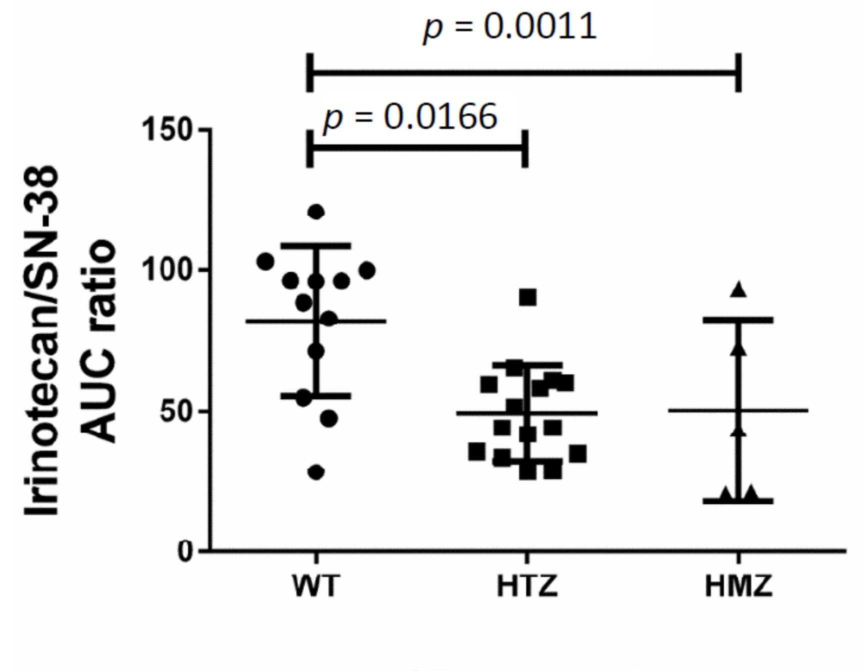

Figure S3. *UGT1A1* variability correlations showing significant correlation between the *UGT1A1* status and the ratio of irinotecan/SN-38 AUCs.

Level	Patients evaluable for radiology	Tumor responses
1	3	1 with SD, 2 with PDs
2	4	4 with SDs
3	3	1 with PR, 2 with PDs
4	3	3 with SDs
5	2	1 with SD, 1 with PD
6	3	3 with PDs
7	3	3 with SDs
8	3	1 with PR, 1 with SD, 1 with PD
9	4	4 with SDs
10	3	1 with PDs, 2 with SDs

Abbreviations: PR=partial response; SD=stable disease; PD=progressive disease

Figure S4. Tumor responses per dose level in RAPIRI trial: 31 evaluable patients. No correlations were observed between tumor responses and dose levels (p=0.65).

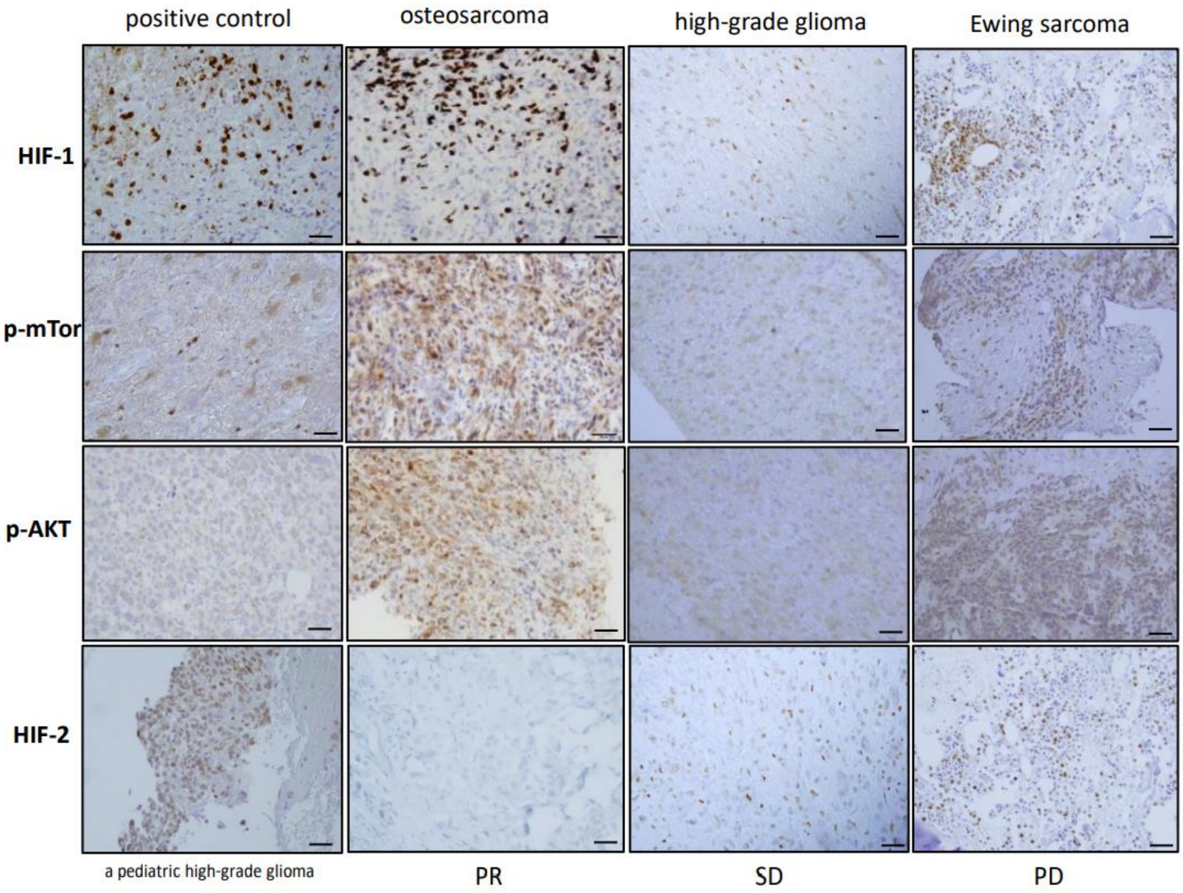

Figure S5. Immunohistochemical profiling analyses according to tumor responses. A positive control (a pediatric high-grade glioma) for all immunohistochemical staining is showed in first column of the figure. In other columns, an example of each response group is presented (scalebar = 20 µm).

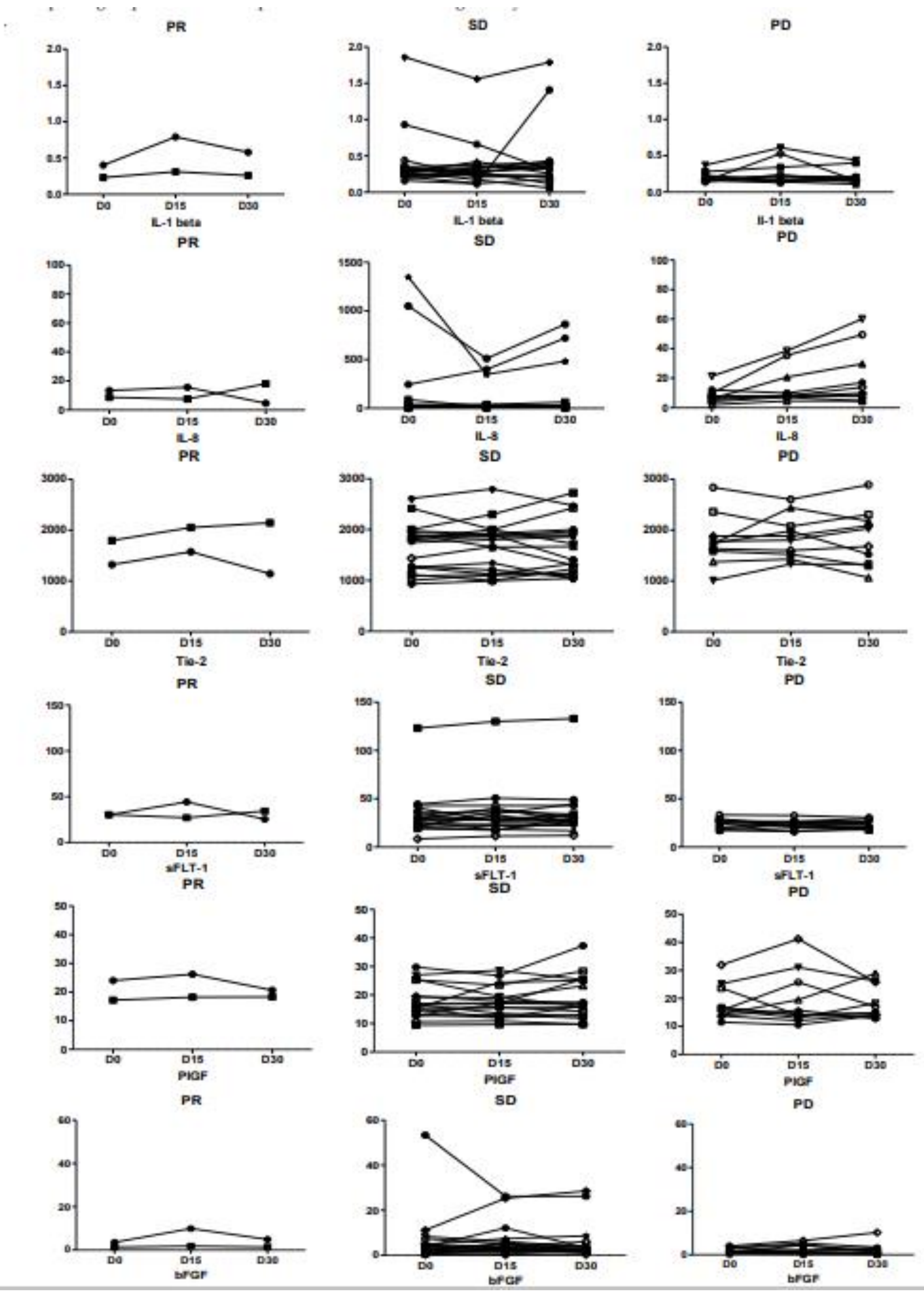

Figure S6. Cytokine evaluation according to response groups: partial response (PR), stable disease (SD) and progressive disease (PD). No statistical significance was observed for IL-1beta, IL-8, Tie-2, sFLT-1, PIGF and bFGF, where almost all response groups had the same plasmatic secretions during first cycle of treatment.
